# Molecularly imprinted polyvinylbenzoic acid based sensor for highly sensitive detection of acute lymphoblastic leukemia biomarker

**DOI:** 10.1007/s00216-026-06602-2

**Published:** 2026-06-13

**Authors:** Ece Ozkan

**Affiliations:** Department of Analytical Chemistry, Faculty of Pharmacy, Ankara Medipol University, Altındağ, Ankara, 06239 Türkiye

**Keywords:** Acute lymphoblastic leukemia, Biomarkers, Molecularly imprinted polymers, Electrochemical sensor

## Abstract

According to data from the World Health Organization, acute lymphoblastic leukemia (ALL) constitutes approximately 80% of leukemia cases in pediatric populations and 20% in adults. The diagnosis of leukemia is predominantly established via hematological analyses and bone marrow biopsies; however, these diagnostic approaches present several inherent limitations and challenges. This study, a novel electrochemical sensor modified with a molecularly imprinted polymer (MIP) for the highly sensitive and selective detection of phosphoenolpyruvate (PEP), a clinically relevant biomarker, with the goal of facilitating early and straightforward diagnosis of acute lymphoblastic leukemia (ALL). A PEP-specific molecularly imprinted polymer (MIP) sensor was fabricated via a UV-induced photopolymerization process, employing *p*-vinylbenzoic acid (VBA) as the functional monomer and ethylene glycol dimethacrylate (EGDMA) as the cross-linking agent. The resulting polymer was subsequently immobilized onto a glassy carbon electrode (GCE). The developed sensor, that was PEP/VBA@MIP-GCE, demonstrated remarkable sensitivity, achieving a limit of detection (LOD) and limit of quantification (LOQ) of 2.24 × 10^–13^ M—6.80 × 10^–13^ M in standard solutions and 1.56 × 10^–13^ M—4.72 × 10^–13^ M in commercial human serum. Furthermore, the sensor exhibited a linear response within the concentration range of 1.0 × 10^⁻12^ M to 1.0 × 10^⁻11^ M. Additionally, high recovery values of 100.36% and 100.97% obtained from commercial human serum samples support the reliability of the sensor. The sensor exhibited a selective analytical performance in the presence of potential interfering substances, underscoring its precision (RSD%: 0.153–1.648) and accuracy (RSD%: 0.048–0.877). This work proposes a novel and first implementation of electrochemical sensing platforms incorporating a molecularly imprinted polymer (MIP) for the detection of PEP, offering a low-cost and efficient strategy suitable for clinical diagnostic applications and pharmaceutical analysis.

**Graphical Abstract:**

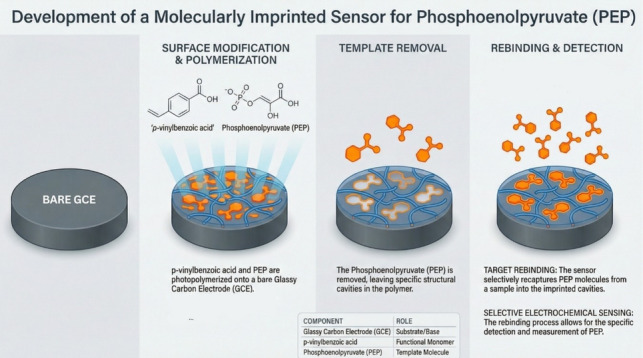

##  Introduction

Leukemia represents a highly aggressive malignancy that significantly affects hematopoietic cells, lymphoid tissues, and bone marrow. Its early detection is often hindered by vague clinical manifestations and the asymptomatic progression of the disease in initial stages. A defining characteristic of leukemia is the dysregulated and excessive proliferation of leukocytes, leading to impaired hematopoietic function [1]. Leukemia classification is determined by the specific type of hematopoietic lineage involved. Acute lymphoblastic leukemia (ALL) is named according to both its origin from lymphoid progenitor cells and its abrupt, rapidly progressing clinical manifestation. These precursor cells are integral to antiviral immune defense, thus the disease is referred to as lymphocytic or lymphoblastic leukemia.

ALL emerges from aberrant proliferation and differentiation processes in lymphoid progenitor cells, culminating in the buildup of malignant populations in the bone marrow and, in some cases, in extramedullary compartments [2]. While the precise mechanisms driving this malignant transformation are not yet fully understood, recent genomic research has focused on uncovering the interplay between environmental exposures and genetic predispositions involved in leukemogenesis [3]. Genomic analyses have significantly advanced the molecular classification of ALL and facilitated the identification of novel disease subtypes [4]. Metabolomics, defined as the systematic and comprehensive analysis of low-molecular-weight metabolites in biological fluids for metabolic phenotyping, has become a powerful approach for elucidating disease-associated pathways and their underlying molecular alterations [5]. Over the past decade, the study of metabolic biomarkers linked to cancer within human tissues and biological fluids has emerged as a pivotal area of research in the field of metabolomics [6]. Metabolomic approaches are now widely employed in the diagnosis and treatment of cancer, as metabolites serve as highly effective biomarkers for disease detection. These biomarkers endogenous molecules that reflect specific physiological or pathological states can be identified in a variety of bodily fluids, offering valuable diagnostic and prognostic information. Moreover, the incorporation of biomarker information facilitates the advancement of personalized treatment approaches customized to the unique characteristics of patients and their specific disease manifestations. This paradigm shift, driven by advancements in metabolomics, underscores the increasing emphasis on human health within contemporary biomedical research [7–9].

Despite the clinical relevance of PEP as a key biomarker for acute lymphoblastic leukemia (ALL), there is a significant lack of studies in the literature regarding its quantitative determination from plasma, with the exception of a few clinical investigations. Accurate measurement of serum PEP levels is of critical clinical importance; however, its endogenous nature and the complex matrix of plasma present significant analytical challenges. Conventional analytical techniques such as chromatography and spectrophotometry, while widely used, are often limited by high cost, time consumption, and complex sample preparation. In contrast, electrochemical sensors have emerged as promising alternatives due to their superior sensitivity, selectivity, reliability, cost-effectiveness, rapid response, ease of use, and portability [10]. In the advancement of electrochemical sensing strategies, the utilization of surface-modified electrodes has become a common approach to enhance analytical sensitivity and target selectivity [11]. A widely adopted strategy involves the modification of electrodes using highly conductive nanomaterials in combination with Molecularly Imprinted Polymer (MIP), which offer both high sensitivity and molecular specificity [12]. MIP is produced via the polymerization of functional monomers in the presence of a target template molecule. For the successful fabrication of a high-performance MIP, a suitable affinity between the functional monomer and the analyte is essential, as the polymerization process is expected to occur around the analyte molecules [13]. Upon removal of the template, specific recognition sites that are complementary in shape and functional groups to the target molecule are formed within the polymer matrix, thereby enabling strong and selective binding [14, 15]. The resulting material can thus be regarded as a biomimetic system that functionally resembles biological recognition elements such as enzymes and antibodies [16]. MIP exhibit high versatility and broad compatibility with diverse analytical platforms, thereby significantly enhancing measurement performance in the design of practical and robust sensing systems [17]. MIP is considered a promising alternative due to their low cost, rapid response, high sensitivity, and compatibility with miniaturized platforms [18]. As three-dimensional polymeric structures, MIP exhibit exceptional and highly selective adsorption properties toward target analytes, even in complex sample matrices [19].

In this work, we introduce a highly innovative MIP-modified electrochemical sensor for the selective and ultrasensitive detection of PEP, a clinically critical biomarker associated with ALL. To the best of our knowledge, this study represents the first report of the quantitative electrochemical determination of PEP, thereby establishing a new analytical paradigm in this field. The proposed sensing platform delivers exceptional selectivity and sensitivity in complex plasma matrices, as validated by DPV, achieving remarkably low detection limits. Importantly, the sensor’s robustness and recovery potential were further demonstrated through the accurate quantification of PEP in commercially available human serum samples. These findings position the developed MIP-based electrochemical platform as a powerful and promising tool for next-generation clinical diagnostics and biomarker monitoring. In conculusion, this work establishes a new benchmark for MIP-based electrochemical sensing and paves the way for its integration into next generation clinical diagnostic systems.

## Experimental

### Chemicals and reagents

The active pharmaceutical ingredients, including phosphoenolpyruvate (PEP), pyruvic acid (PA), α-ketoisovaleric acid (KIV), and lysophosphatidylcholine (LPC), were sourced from Sigma-Aldrich. Additional reagents obtained from the same supplier were employed for various experimental purposes as detailed below. Methanol (MeOH; ≥ 99.8%) was used in the preparation of stock solutions for drug compounds, whereas acetonitrile (ACN; ≥ 99.9%) served as a protein-precipitating agent during serum sample processing. Potassium ferrocyanide (K₄[Fe(CN)₆]; ≥ 98.5%) and potassium ferricyanide (K₃[Fe(CN)₆]; ≥ 99.0%) were utilized as redox probes in electrochemical analyses. Monomer and crosslinking agents such as ethylene glycol dimethacrylate (EGDMA; > 98.0%), 2-hydroxyethyl methacrylate (HEMA; ≥ 99.0%), vinylbenzoic acid (VBA; ≥ 97.0%), and the photoinitiator 2-hydroxy-2-methylpropiophenone (> 97.0%) were employed for polymerization processes. For cleaning and regeneration purposes, sodium hydroxide (> 97.0%), ethanol (EtOH; 99.8%), hydrochloric acid (HCl), and acetic acid (AcOH; ≥ 99.8%) were used. To evaluate selectivity and potential matrix interference, solutions containing ascorbic acid (AA), dopamine (DOP), uric acid (UA; ≥ 99.0%), paracetamol (PAR), magnesium chloride (MgCl₂; ≥ 99.0%), sodium sulfate (Na₂SO₄; ≥ 99.0%), and potassium nitrate (KNO₃; ≥ 99.0%) were prepared. In addition, synthetic human serum (origin: male AB plasma, USA) was used for the simulation of biological sample analysis. All chemical reagents and stock solutions were stored at approximately 4 °C to maintain their chemical stability and prevent degradation throughout the study period.

### Equipment and chemicals

Electrochemical analyses, including differential pulse voltammetry (DPV), cyclic voltammetry (CV), and electrochemical impedance spectroscopy (EIS), were carried out with an Ivium CompactStat potentiostat (Ivium Technologies, Netherlands) operated through the IviumSoft software interface. The sensing platform consisted of a glassy carbon electrode (GCE, 3.0 mm diameter) modified with a molecularly imprinted polymer (MIP) as the working electrode, accompanied by an Ag/AgCl (3 M KCl) reference electrode and a platinum wire counter electrode in a standard three-electrode setup (IN, USA). All chemical reagents were precisely measured using an analytical balance (Ohaus Corporation, China). Throughout the fabrication process, a thermo-shaker (Biosan TS-100) was employed for controlled agitation, while a vortex mixer (ISOLAB Laborgeräte GmbH, Germany) and an ultrasonic bath (JP Selecta, Spain) were used to promote homogeneity of the polymer mixtures. The MIP films were polymerized via UV irradiation (100 W, 365 nm). The surface morphology of the resulting films was analyzed using scanning electron microscopy (SEM) on a TESCAN GAIA 3 system (TESCAN, Czechia).

The active pharmaceutical ingredients including PEP, pyruvic acid (PA), α-ketoisovaleric acid (KIV), and lysophosphatidylcholine (LPC) were obtained from Sigma-Aldrich. All additional reagents were also supplied by Sigma-Aldrich and utilized according to their specific purposes. Methanol (MeOH, ≥ 99.8%) was employed for the preparation of stock drug solutions, while acetonitrile (ACN, ≥ 99.9%) was used for serum protein precipitation. Potassium ferrocyanide (K₃[Fe(CN)₆], ≥ 98.5%) and potassium ferricyanide (K₄[Fe(CN)₆], ≥ 99.0%) served as redox probes in electrochemical measurements. For polymerization procedures, ethylene glycol dimethacrylate (EGDMA, > 98.0%), 2-hydroxyethyl methacrylate (HEMA, ≥ 99.0%), 2-hydroxy-2-methylpropiophenone (> 97.0%), and vinylbenzoic acid (VBA, ≥ 97.0%) were utilized. Sodium hydroxide (> 97%), ethanol (EtOH, 99.8%), hydrochloric acid (HCl), and acetic acid (AcOH, ≥ 99.8%) were used in the preparation of regeneration and washing solutions. Ascorbic acid (AA), dopamine (DOP), uric acid (UA, ≥ 99.0%), paracetamol (PAR), magnesium chloride (MgCl₂, ≥ 99.0%), sodium sulfate (Na₂SO₄, ≥ 99.0%), and potassium nitrate (KNO₃, ≥ 99.0%) were employed to prepare interference solutions. Synthetic human serum (heat inactivated, from human male AB plasma, USA origin, sterile-filtered) by Sigma-Aldrich, which was used for biological sample analysis. All chemicals and prepared stock solutions were stored at approximately 4 °C to maintain their stability and prevent degradation.

### Fabrication of MIP and NIP modified sensor

**Scheme 1:**
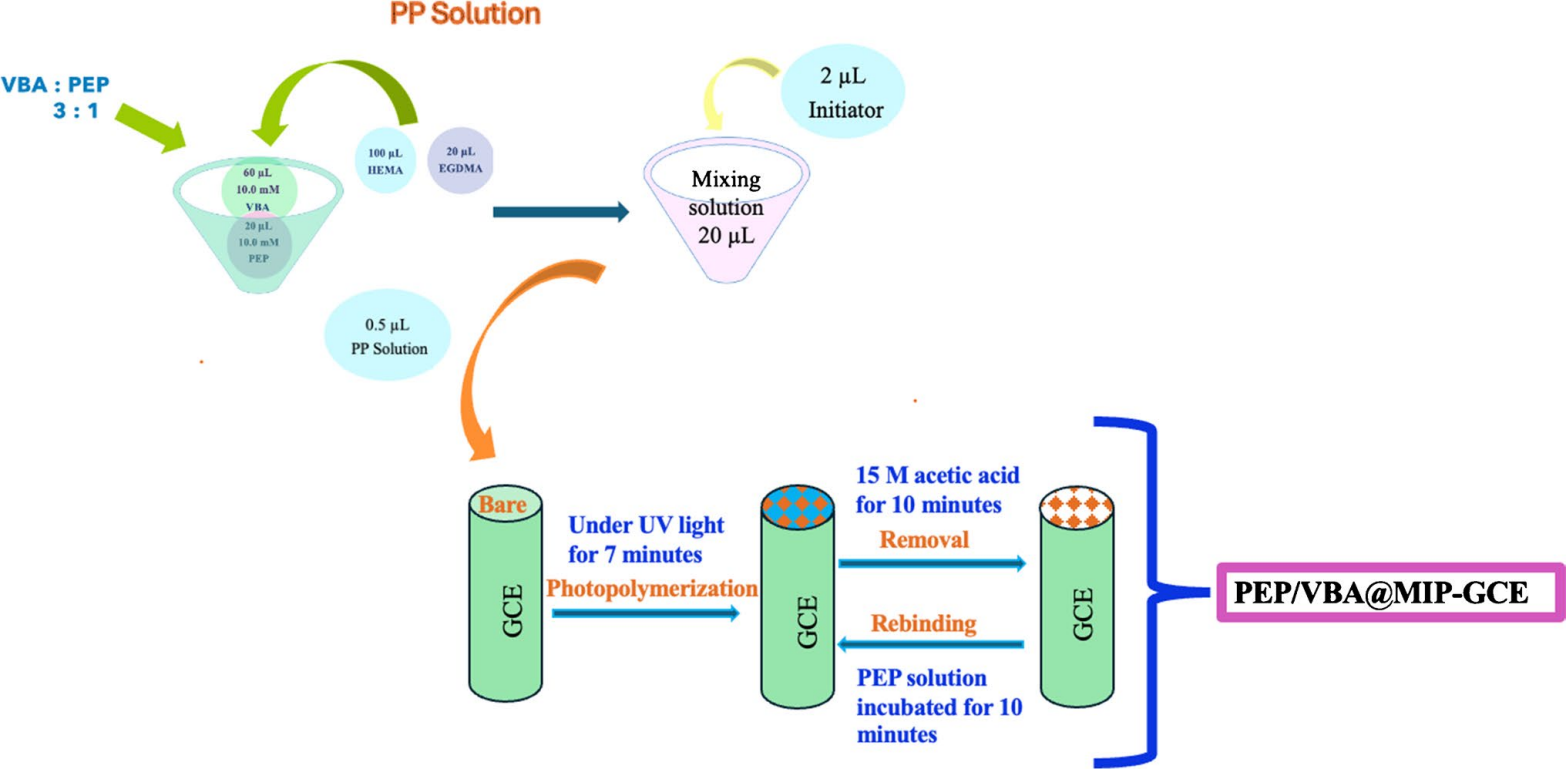
Fabrication steps of PEP/VBA@MIP-GCE sensors

The PEP-selective electrochemical sensor was fabricated through a photopolymerization (PP) approach (Scheme 1). For this purpose, a polymerization mixture containing VBA and PEP at a molar ratio of 3:1 was initially prepared. Subsequently, 100 µL of HEMA and 20 µL of EGDMA were introduced, and the solution was stirred for 5 min at room temperature to achieve uniform dispersion. Into 20 µL of this mixture, 2-hydroxy-2-methylpropiophenone (2 µL) was incorporated as the photoinitiator and vortexed for 1 min. Afterwards, 0.5 µL of the resulting polymeric solution was carefully dropped onto the GCE surface. The polymerization process was carried out under UV irradiation (365 nm, 100 W) for 7 min, followed by standing at room temperature for an additional 7 min. Removal of the template molecule was achieved by immersing the modified electrode in 15 M acetic acid for 10 min while shaking at 650 rpm and 25 °C. For the rebinding step, the GCE was exposed to a 10⁻⁹ M PEP solution and incubated for 10 min under identical shaking conditions. Prior to each electrochemical measurement, the PEP/VBA@MIP-GCE electrode was rinsed with distilled water for 30 s to ensure cleanliness and reusability. A corresponding non-imprinted polymer (PEP/VBA@NIP-GCE) was prepared using the same PP procedure, excluding the PEP template, to serve as a control. Electrochemical characterization (DPV, CV, and EIS) was conducted using a 5 mM [Fe(CN)₆]3⁻/4⁻ redox couple as the probing solution.

### Preparation of synthetic human serum sample

A 0.1 mM serum standard solution was prepared by mixing 3600 μL of a synthetic serum sample (previously stored at − 20 °C) with 5400 μL of acetonitrile and 1000 μL of PEP stock solution. The resulting mixture was sonicated for 15 min to facilitate complete homogenization and analyte release, then centrifuged at 5000 rpm for 20 min at 25 °C. The resulting clear supernatant was carefully separated and transferred into an electrochemical cell for subsequent analysis. To assess the sensor’s applicability in complex biological media, recovery experiments were performed using the standard addition approach combined with DPV.

## Results and discussion

### Optimization steps of photopolymerization conditions

**Fig. 1:**
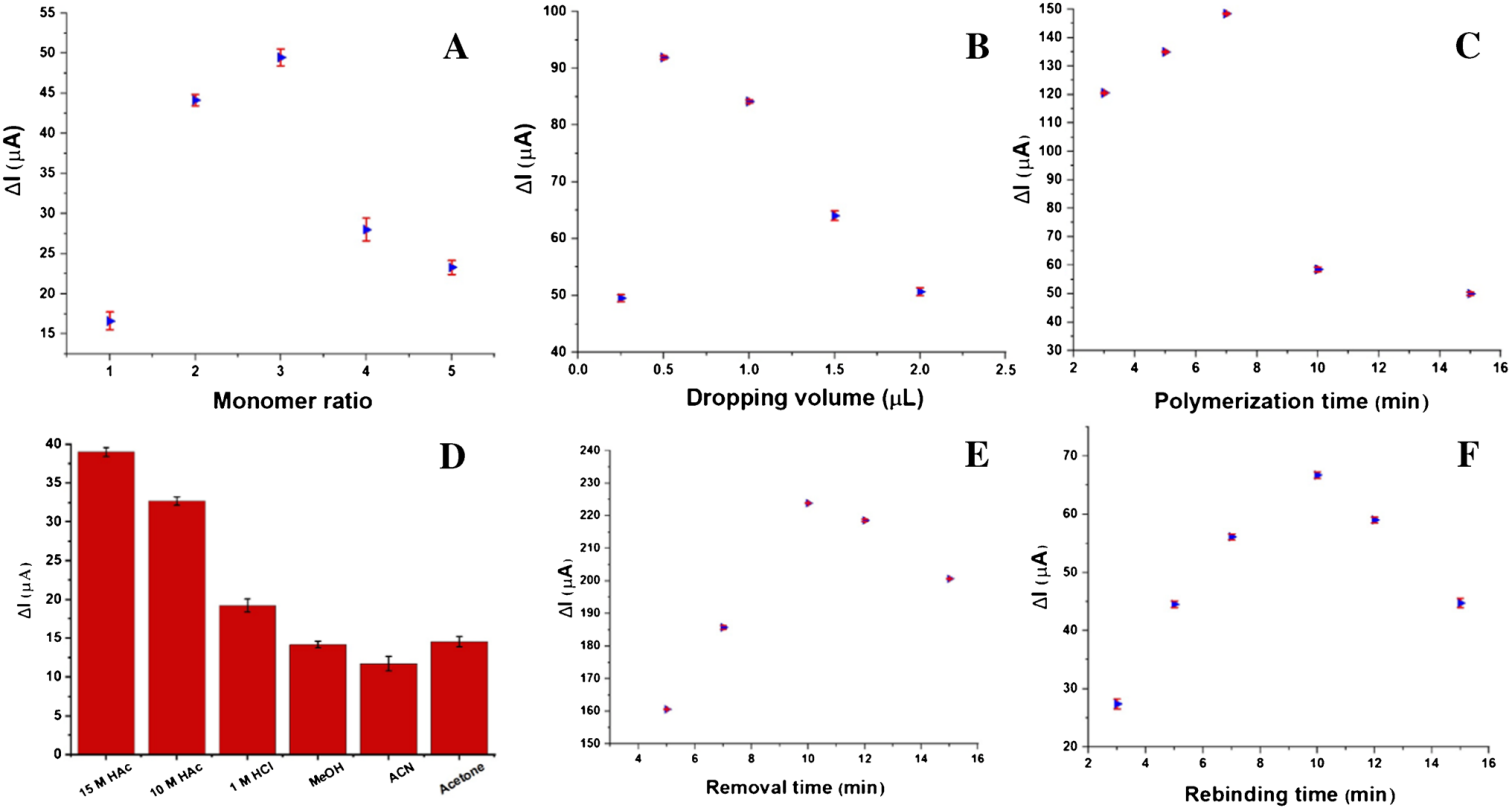
Variation of ΔI with respect to (**A**) monomer-to-template ratio, (**B**) deposition volume, (**C**) polymerization duration, (**D**) type of removal solvent, (**E**) extraction period, and (**F**) rebinding duration, measured in a 5 mM [Fe(CN)₆]^3^⁻/.^4^⁻ electrolyte solution (n = 3)

Sensor optimization was systematically carried out by investigating key parameters including the monomer-to-template ratio, the applied volume of the polymerization mixture, the duration of the photopolymerization process, as well as the efficiency of template removal and subsequent rebinding (Fig. 1). During the optimization studies, the binding performance of the PEP/VBA@MIP-GCE sensor was evaluated using 1.0 × 10⁻^1^⁰ M PEP as the model analyte. At this critical stage directly associated with the analytical performance the difference in current values after removal and rebinding, denoted as ΔI, is taken into account.

#### Functional monomer: template ratio

To determine the optimal composition for sensor fabrication, various monomer-to-template (VBA:PA) ratios were evaluated at weight ratios of 1:1, 2:1, 3:1, 4:1, and 5:1 (Fig. 1A). Among the tested conditions, the ratio of 3:1 (w/w) was identified as the most effective for constructing the PEP/VBA@MIP-GCE sensor, yielding the highest binding response.

#### Dropping volume

Optimization of the PP solution volume was carried out by drop-casting varying volumes, ranging from 0.25 μL to 2.00 μL, onto the surface of the GCE. The greatest change in current measured before and after template molecule removal was observed at a PP solution volume of 0.5 μL, indicating this as the optimal deposition volume (Fig. 1B).

#### Photopolymerization time

The duration of UV exposure for polymerization was examined at intervals ranging from 3 to 15 min, following the deposition of the PP solution onto the electrode surface. Optimal formation of the MIP film, characterized by stable and reproducible sensor responses, was achieved with a UV irradiation time of 7 min (Fig. 1C).

#### Removal solution and time

To remove the template molecule from the PEP/VBA@MIP-GCE sensor, various solvents including HAc (15 M and 10 M), methanol (MeOH), hydrochloric acid (1 M HCl), acetonitrile (ACN), and acetone were tested by incubating the modified electrode in each solution on a shaker at 650 rpm and 25 °C for 10 min. Among these, 15 M HAc demonstrated the most efficient and consistent extraction of the template molecule, as evidenced by the greatest change in current following polymerization (Fig. 1D).

Subsequently, the optimal extraction time using 15 M HAc was determined by immersing the PEP/VBA@MIP-GCE sensor for 5, 7, 10, 12, and 15 min under the same shaking conditions. The most significant increase in peak current, indicative of effective template removal, was observed at 10 min (Fig. 1E). Therefore, the most effective template removal was achieved using 15 M HAc with a 10 min period.

#### Rebinding time

The effect of incubation time on the rebinding efficiency of the PEP/VBA@MIP-GCE sensor was assessed using a 1.0 × 10⁻^1^⁰ M PEP solution. The sensor was incubated under shaking conditions (650 rpm, 25 °C) for 3, 5, 7, 10, and 15 min. The amount of PEP rebinding increased progressively up to 10 min, after which a slight decline was noted. Consequently, an incubation time of 10 min was identified as optimal for efficient target molecule rebinding (Fig. 1F).

### Physical and electrochemical characterizations

**Fig. 2:**
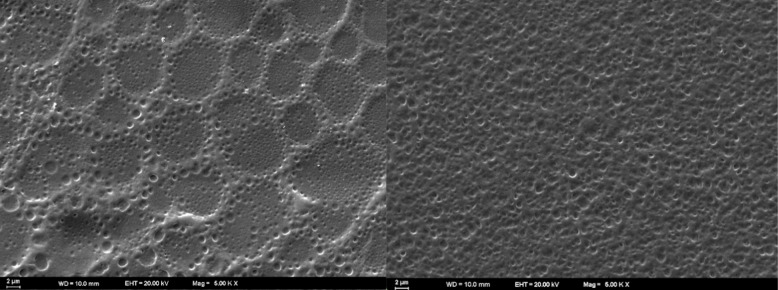
Surface morphology characterization of the modified electrodes, showing SEM images of (A) MIP and (B) NIP films prepared under identical conditions

Surface morphologies of the PEP/VBA@MIP-GCE and PEP/VBA@NIP-GCE sensors were examined using SEM imaging (Figs. 2A and B). The MIP-modified surface (Fig. 2A) displayed a distinctly rough and porous morphology, indicative of the formation of specific recognition cavities resulting from the molecular imprinting process. These features reflect the presence of binding sites tailored for the target molecule. In contrast, the NIP-modified surface (Fig. 2B) exhibited a comparatively smooth and homogeneous texture, with minimal surface irregularities, characteristic of non-imprinted polymers that lack selective binding sites.

The electrochemical characterization of the PEP/VBA@MIP-GCE sensor was performed using CV, DPV, and EIS. CV and DPV were utilized to evaluate the oxidation and reduction peak currents of a standard redox probe at each fabrication stage: bare GCE, after polymer deposition (PP), after template removal, and following template rebinding. EIS measurements assessed the charge transfer resistance (Rct) of the electrode surface throughout these stages.

**Fig. 3:**
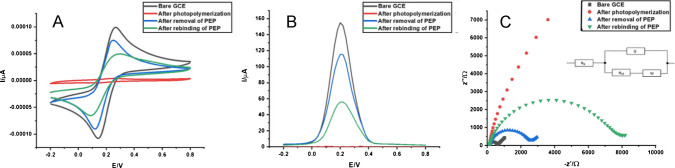
(A) Cyclic voltammograms obtained at a scan rate of 50 mV s⁻^1^ in 5 mM [Fe(CN)₆]^3^⁻/^4^⁻ solution recorded for: (1) bare GCE, (2) electrode after photopolymerization, (3) MIP-modified electrode following PEP extraction, and (4) MIP sensor after a 5 min rebinding process with 1.0 × 10⁻^1^⁰ M PEP. (B) Differential pulse voltammograms measured under the same conditions for the same sequence of electrodes. (C) Corresponding Nyquist plots obtained in 5 mM [Fe(CN)₆]^3^⁻/^4^⁻ solution illustrating the impedance responses at each modification step

In CV analysis (Fig. 3A), the bare GCE exhibited the highest oxidation and reduction peak currents, indicative of a clean surface facilitating rapid electron transfer. After polymerization, the peak currents significantly decreased due to the insulating polymer film that hindered electron transfer. Template removal partially restored the peak currents, reflecting the formation of recognition sites that allowed electron transport. Upon rebinding of PEP, peak currents decreased again, consistent with occupation of the imprinted cavities restricting electron flow.

DPV measurements (Fig. 3B) corroborated these findings, showing analogous trends in peak current changes corresponding to each fabrication step, thereby confirming the sensor’s selective binding capability.

EIS data represented as Nyquist plots (Fig. 3C) revealed for bare GCE, after PP, after template removal, and after rebinding, respectively. These results align with CV and DPV data, where polymer formation increased resistance, template removal decreased it, and rebinding partially increased resistance due to the restored molecular recognition sites. Low charge transfer resistance (Rct) values of 163 Ω were obtained for the bare GC sensor, while this value increased to 7518 Ω after polymerization. A decrease in Rct (1035 Ω) was observed after removal, but a significant increase in Rct (1742 Ω) was achieved by reconnecting the PEP to the sensor surface.

### Determination of SEL using SEL@4-ABA/MIP/GCE sensor

**Fig. 4:**
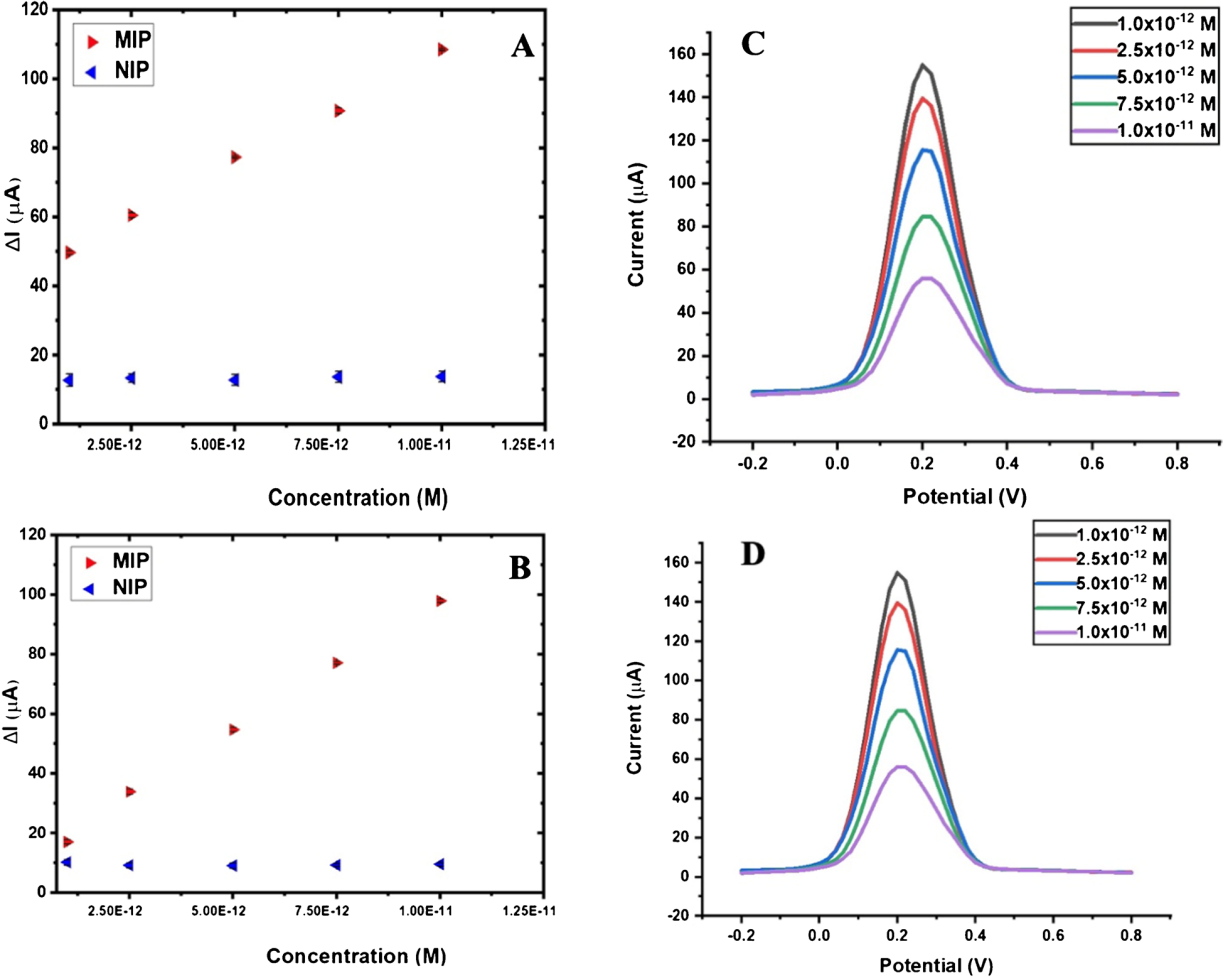
The calibration curve of PEP on PEP/VBA@MIP-GCE and PEP/VBA@NIP-GCE in A) standard solution and B) synthetic human serum; DPV voltammograms after rebinding of different SEL concentrations in C) standard solution and D) commercial human serum

Determination of PEP was evaluated by calculating the ΔI values after rebinding of selected PEP concentrations using the PEP/VBA@MIP-GCE sensor. For the PEP/VBA@MIP-GCE sensor, the obtained ΔI values were plotted against PEP concentrations, revealing a linear range between 1.0 × 10⁻^12^ M and 1.0 × 10⁻^11^ M (Fig. 4A). Conversely, when the NIP-based GCE sensor was evaluated across the identical concentration range, it failed to demonstrate a notable or linear electrochemical response toward PEP, primarily due to its insufficient molecular recognition capability (Fig. 4A). The differential pulse voltammetry (DPV) voltammograms obtained after rebinding PEP within the concentration range of 1.0 × 10⁻^12^ M to 1.0 × 10⁻^11^ M are shown in Fig. 4B. Regression data, presented in Table 1, yielded the calibration equation: ΔI (μA) = 6.0 × 10^12^ (μA/M) × C (M) + 44.05 (r = 0.998). Method sensitivity was evaluated in accordance with ICH guidelines by calculating the limit of detection (LOD) and limit of quantification (LOQ). The LOD and LOQ were determined using the formulas 3.3σ/slope and 10σ/slope, respectively, yielding values of 2.24 × 10^–13^ M and 6.80 × 10^–13^ M.

**Table 1:** Analytical performance parameters of PEP/VBA@MIP-GCE sensor in standard and synthetic human serum solutions

	PEP (Standard solution)	PEP (Synthetic human serum)
Linearity range (M)	1.0 × 10^–12^ – 1.0 × 10^–11^	1.0 × 10^–12^ – 1.0 × 10^–11^
Slope (μA M^−1^)	6.0 × 10^+12^	9.0 × 10^+12^
Intercept (μA)	44.054	9.985
Determination coefficient	0.998	0.998
LOD (M)	2.24 × 10^–13^	1.56 × 10^–13^
LOQ (M)	6.80 × 10^–13^	4.72 × 10^–13^
The precision of peak current (Interday, n = 5) (RSD %)	0.153	0.565
The precision of peak current (Intraday, n = 5) (RSD %)	0.624	1.648
The accuracy of peak current (Interday, n = 5) (Bias %)	0.048	0.275
The accuracy of peak current (Intraday, n = 5) (Bias %)	0.089	0.877

### Application in synthetic human serum samples

Following the validation of the developed sensor in standard solutions, its performance was further evaluated in biological matrices. For this purpose, a spiked human serum sample was prepared in accordance with the procedure detailed in Sect. "Preparation of synthetic human serum sample" and employed for the quantification of PEP using the PEP/VBA@MIP-GCE sensor. The sensor demonstrated a linear electrochemical response across the concentration range of 1.0 × 10⁻^12^ M to 1.0 × 10⁻^11^ M (Fig. 4C). The corresponding calibration equation was determined to be: ΔI (μA) = 9.0 × 10^12^ (μA/M) × C (M) + 9.99, with a correlation coefficient (r) of 0.998. DPV corresponding to PEP rebinding within this linear range are illustrated in Fig. 4D. Comprehensive regression data, including the calculated LOD: 1.56 × 10^–13^ M and LOQ: 4.72 × 10^–13^ M, are summarized in Table 1.

To assess the sensor's selectivity in complex biological media, recovery tests were subsequently performed in the human serum matrix by introducing known two concentrations of PEP which were 2.5 × 10⁻^12^ M and 5.0 × 10⁻^12^ M. The method yielded an average recovery rate of 100.36% and 100.97%, with RSD of 0.90% and 0.24%, respectively (Table 2), thereby verifying the high selectivity and reliability of the PEP/VBA@MIP-GCE sensor under real-sample conditions.

**Table 2:** Results of recovery experiments performed on synthetic human serum samples

	**Serum Sample 1**	**Serum Sample 2**
Spiked amount (M)	2.50 × 10^–12^	5.00 × 10^−12^
Found amount (M)*	2.49 × 10^–12^	4.99 × 10^–12^
Average recovery (%)*	100.36	100.97
RSD% of recovery	0.90	0.24
Bias%	−0.36	−0.97

The proposed sensor demonstrates competitive analytical performance compared to previously reported methods, offering a wide linear dynamic range, low detection limit, and satisfactory real-sample applicability (Table 3). Unlike traditional techniques, it provides a simple, cost-effective, and environmentally friendly alternative by eliminating complex procedures and hazardous reagents. Its excellent selectivity, reproducibility, and stability further emphasize its suitability for practical applications.

**Table 3:** Comparison of currently available studies in the literature to this sensor

Method	Sample	LOD	LOQ	Linear Range	Recovery %	Ref
HILIC-MS/MS	Plasma Serum	0.3 μg/L 1 μg/L	1 μg/L 10 μg/L	1–250 μg/L 10–500 μg/L	100.0% 107.2%	[20]
IC-MS/MS	P. pastoris cell	-	-	-	> 70%	[21]
GC–IDMS	Algal cells	10 pmol	-	10–1000 pmol	355.6%	[22]
ESI–MS/MS	Escherichia coli	50 ng/mL	–	1900–1 ng/mL	-	[23]
HILIC–ESI–MS/MS	Alkaline and Acid mobile phases	86.5 nM 532.7 nM	-	50–5000 nM 500–5000 nM	-	[24]
LC–MS/MS	Escherichia coli	3.8 nM	13.1 nM	5–10 nM	-	[25]
LC–MS	Human platelets		0.49 pmol	10–500 ng/ mL	-	[26]
MIP-based electrochemistry	Serum	1.56 × 10^−13^ M	4.72 × 10-^13^ M	1.0 × 10^–12^–1.0 × 10^–11^ M	100.36% 100.97%	This work

### Interference study

To evaluate the selectivity and practical applicability of the fabricated PEP/VBA@MIP-GCE sensor, its response was investigated in the presence of potential interfering substances commonly found in biological matrices. Representative compounds such as K⁺, Cl⁻, dopamine, and paracetamol were selected due to their likelihood of coexisting with PEP and potentially influencing the sensor signal. Interference tests were carried out using solutions in which the concentrations of these species were either equivalent to or tenfold higher than that of PEP. The obtained results, summarized in Table 4 as percent recovery and RSD, indicate minimal variation in sensor response. These findings demonstrate that the presence of interfering substances does not significantly compromise the selectivity or reliability of the PEP/VBA@MIP-GCE sensor.

**Table 4:** Effect of potential interferents on the quantification of PEP

	**1:1**		**1:10**	
Interfering Agents (IA)	**Recovery(%)**	**RSD (%)**	**Recovery (%)**	**RSD (%)**
K^+^	**100.34**	**0.27**	**100.52**	**0.23**
NO_3_^−^	**100.34**	**0.27**	**100.52**	**0.23**
Mg^+^	**100.01**	**0.46**	**101.02**	**0.11**
Cl_2_^−^	**100.01**	**0.46**	**101.02**	**0.11**
Na^+^	**100.41**	**0.34**	**101.29**	**0.53**
SO_4_^2−^	**100.41**	**0.34**	**101.29**	**0.53**
DOP	**99.72**	**0.57**	**99.40**	**0.49**
AA	**99.19**	**0.55**	**99.49**	**0.16**
UA	**99.93**	**0.48**	**98.63**	**0.44**
PAR	**99.34**	**0.98**	**98.81**	**0.52**

### Imprinting factor study

As highlighted throughout the study, the key feature that distinguishes the proposed sensor from previously reported systems is its exceptional selectivity. The observed selectivity is attributed to the creation of molecularly imprinted cavities that possess complementary shapes and functional groups specifically tailored to the target analyte. However, structurally similar compounds may still pose a risk of non-specific binding, potentially affecting the sensor’s selectivity. To evaluate this, structurally analogous molecules, pyruvic acid, α-ketoisovaleric acid, and lysophosphatidylcholine, were chosen for imprinting factor (IF) analysis. The results of the selectivity analysis demonstrated satisfactory performance, as the proposed sensor exhibited enhanced recognition toward PEP in the presence of potential interfering species. The ΔI values were determined to assess the binding response of each compound on both the MIP and NIP surfaces. The relative imprinting factor (IF'), defined as the ratio of IF values between MIP and NIP, was used as an indicator of selectivity and is expected to exceed 1. As presented in Table 5, all calculated IF values were above 3, confirming the strong recognition capability of the sensor toward PEP over structurally related interferents. Additionally, the t-test performed for PEP yielded a p-value of 1.55 × 10⁻^1^⁰. Since p < 0.05, the difference between the MIP and NIP values was found to be significant. Furthermore, since the p-values for LPC, PA, and KIV were 0.05, the difference between the MIP and NIP sensor results for these molecules was found to be insignificant.

**Table 5:** The IF’ values for PEP and similar molecules

	MIP		NIP		
	ΔI/µA	IF(MIP)	ΔI/µA	IF(NIP)	IF'(MIP/NIP)
PEP	77.392		12.830		
PA	13.116	5.901	9.70	1.323	4.461
KIV	13.548	5.712	10.50	1.221	4.677
LPC	12.721	6.084	10.693	1.200	5.071

## Conclusion

This study reports the development and effective application of a MIP-based electrochemical sensor specifically designed for the sensitive detection of PEP within biological matrices. The MIP recognition layer was prepared via photopolymerization of PVA, providing excellent selectivity towards PEP even in the presence of structurally related ALL biomarkers such as PA, KIV, and LPC. The sensor exhibited a robust linear correlation between DPV signals and the logarithmic concentration of PEP within the range of 1.0 × 10⁻^12^ M to 1.0 × 10⁻^11^ M (r = 0.998). Remarkably, the LOD was determined to be 2.24 × 10^–13^ M, surpassing the sensitivity of previously reported methods. Analytical validation was performed using commercial human serum samples spiked with PEP, where the sensor achieved recovery rates between 0.90% and 0.24%, confirming its practical applicability and reliability in complex biological environments. To the best of our knowledge, this study constitutes the first report demonstrating an electrochemical sensor modified with a PVA-based MIP for the detection of PEP. The sensor stands out for its ease of fabrication, low production cost, and exceptional analytical performance, offering excellent sensitivity and selectivity. Given these advantages, the PEP/VBA@MIP-GCE platform demonstrates strong potential as a versatile tool for clinical diagnostics and pharmaceutical quality control. Furthermore, this study lays the groundwork for future investigations into the selective recognition of pharmacologically relevant molecules across diverse sample types, including biological fluids, pharmaceutical formulations, and environmental systems.

Targeting ALL, one of the most critical and widespread cancer types worldwide, this study introduces a breakthrough approach for the detection of its clinically relevant biomarker, PEP. As the first and, to the best of our knowledge, only report enabling precise, accurate, selective and sensitive electrochemical quantification of PEP, this work addresses a significant gap in the literature. The ability to directly measure PEP levels in plasma offers a powerful tool for improved diagnosis and therapeutic monitoring. Overall, this study not only establishes a new benchmark in MIP-based electrochemical sensing but also holds strong potential to drive future advancements in biomarker-based clinical diagnostics.

## Data Availability

All data has been provided in the manuscript.

## References

[1] Danesh NM, Lavaee P, Ramezani M, Abnous K, Taghdisi SM. Targeted and controlled release delivery of daunorubicin to T-cell acute lymphoblastic leukemia by aptamer-modified gold nanoparticles. Int J Pharm. 2015;489(1):311–7. 25936625 10.1016/j.ijpharm.2015.04.072

[2] Paul S, Kantarjian H, Jabbour EJ. Adult Acute Lymphoblastic Leukemia. Mayo Clin Proc. 2016;91(11):1645–66. 27814839 10.1016/j.mayocp.2016.09.010

[3] Pui C-H, Relling MV, Downing JR. Acute lymphoblastic leukemia. N Engl J Med. 2004;350(15):1535–48. 15071128 10.1056/NEJMra023001

[4] Wu C, Li W. Genomics and pharmacogenomics of pediatric acute lymphoblastic leukemia. Crit Rev Oncol Hematol. 2018;126:100–11. 29759551 10.1016/j.critrevonc.2018.04.002

[5] Iliou A, Chekmeneva E, Pinto RC, Koukouzeli FE, Ntounias Y, Georgakopoulou K, et al. A Multiomic Approach Integrating Genomic and Metabolomic Data Highlights Colorectal Cancer Pathways. J Proteome Res. 2026;25(2):578–88. 41603468 10.1021/acs.jproteome.5c00459 PMC12887983

[6] Spratlin JL, Serkova NJ, Eckhardt SG. Clinical Applications of Metabolomics in Oncology: A Review. Clin Cancer Res. 2009;15(2):431–40. 19147747 10.1158/1078-0432.CCR-08-1059 PMC2676437

[7] Nemutlu E, Zhang S, Gupta A, Juranic NO, Macura SI, Terzic A, et al. Dynamic Phosphometabolomic Profiling of Human Tissues and Transgenic Models By O-18-Assisted P-31 Nmr and Mass Spectrometry. Amer Physiological Soc; 2012. 10.1152/physiolgenomics.00152.2011 PMC3339850 22234996

[8] Nemutlu E, Zhang S, Juranic NO, Terzic A, Macura S, Dzeja P. 18O-Assisted Dynamic Metabolomics for Individualized Diagnostics and Treatment of Human Diseases. 2012. 10.3325/cmj.2012.53.529 PMC3541579 23275318

[9] Nemutlu E, Juranic N, Zhang S, Ward LE, Dutta T, Nair KS, et al. Electron spray ionization mass spectrometry and 2D 31P NMR for monitoring 18O/16O isotope exchange and turnover rates of metabolic oligophosphates. Anal Bioanal Chem. 2012;403(3):697–706. 22427058 10.1007/s00216-012-5899-5 PMC3349359

[10] Jean-Michel K, Petr Z, Sibel AO. Electroanalytical Techniques Most Frequently Used in Drug Analysis. Monographs in Electrochemistry ISBN: 9783662471371: Springer Berlin Heidelberg; 2015.

[11] Ciani I, Schulze H, Corrigan DK, Henihan G, Giraud G, Terry JG, et al. Development of immunosensors for direct detection of three wound infection biomarkers at point of care using electrochemical impedance spectroscopy. Biosens Bioelectron. 2012;31(1):413–8. 22137369 10.1016/j.bios.2011.11.004

[12] Han S, Li B, Song Z, Pan S, Zhang Z, Yao H, et al. A kanamycin sensor based on an electrosynthesized molecularly imprinted poly-: O -phenylenediamine film on a single-walled carbon nanohorn modified glassy carbon electrode. Analyst. 2017;142(1):218–23. 10.1039/c6an02338j 27922643

[13] Jara-Cornejo E, Khan S, Vega-Chacón J, Wong A, da Silva Neres LC, Picasso G, et al. Biomimetic material for quantification of methotrexate using sensor based on molecularly imprinted polypyrrole film and MWCNT/GCE. Biomimetics. 2023;8(1):77. 10.3390/biomimetics8010077 PMC9944472 36810408

[14] Tarannum N, Hendrickson OD, Khatoon S, Zherdev AV, Dzantiev BB. Molecularly imprinted polymers as receptors for assays of antibiotics. Crit Rev Anal Chem. 2020;50(4):291–310. 31210058 10.1080/10408347.2019.1626697

[15] Frasco MF, Truta LAANA, Sales MGF, Moreira FTC. Imprinting technology in electrochemical biomimetic sensors. Sensors. 2017;17(3):523. 10.3390/s17030523 PMC5375809 28272314

[16] Menger M, Yarman A, Erdőssy J, Yildiz HB, Gyurcsányi RE, Scheller FW. MIPs and aptamers for recognition of proteins in biomimetic sensing. Biosensors. 2016;6(3):35. 10.3390/bios6030035 PMC5039654 27438862

[17] Golijanin J, Lee DH, Li RY, Ahmadi S. Molecular imprinting polymer-based sensing of neonicotinoids. Sensors. 2025;25(23):7251. 10.3390/s25237251 PMC12694491 41374625

[18] Song H, Wang Y, Zhang L, Tian L, Luo J, Zhao N, et al. An ultrasensitive and selective electrochemical sensor for determination of estrone 3-sulfate sodium salt based on molecularly imprinted polymer modified carbon paste electrode. Anal Bioanal Chem. 2017;409(27):6509–6519. 10.1007/s00216-017-0598-x 28889259

[19] Guo X, Kulkarni A, Doepke A, Halsall HB, Iyer S, Heineman WR. Carbohydrate-Based Label-Free Detection of Escherichia coli ORN 178 Using Electrochemical Impedance Spectroscopy. Anal Chem. 2012;84(1):241–6. 22035288 10.1021/ac202419u

[20] Shen D, Zhu Y, Mao J, Lin R, Jiang X, Liang L, et al. Highly sensitive and accurate measurement of underivatized phosphoenolpyruvate in plasma and serum via EDTA-facilitated hydrophilic interaction liquid chromatography–tandem mass spectrometry. Talanta. 2024;275:126134. 38692044 10.1016/j.talanta.2024.126134

[21] Si-Hung L, Troyer C, Causon T, Hann S. Sensitive quantitative analysis of phosphorylated primary metabolites using selective metal oxide enrichment and GC- and IC- MS/MS. Talanta. 2019;205:120147. 10.1016/j.talanta.2019.120147 31450417

[22] Vielhauer O, Zakhartsev M, Horn T, Takors R, Reuss M. Simplified absolute metabolite quantification by gas chromatography-isotope dilution mass spectrometry on the basis of commercially available source material. J Chromatogr, B: Anal Technol Biomed Life Sci. 2011;879(32):3859–70. 10.1016/j.jchromb.2011.10.036 22100557

[23] Bajad SU, Lu W, Kimball EH, Yuan J, Peterson C, Rabinowitz JD. Separation and quantitation of water soluble cellular metabolites by hydrophilic interaction chromatography-tandem mass spectrometry. J Chromatogr A. 2006;1125(1):76–88. 16759663 10.1016/j.chroma.2006.05.019

[24] Teleki A, Sánchez-Kopper A, Takors R. Alkaline conditions in hydrophilic interaction liquid chromatography for intracellular metabolite quantification using tandem mass spectrometry. Anal Biochem. 2015;475:4–13. 25600449 10.1016/j.ab.2015.01.002

[25] Luo B, Groenke K, Takors R, Wandrey C, Oldiges M. Simultaneous determination of multiple intracellular metabolites in glycolysis, pentose phosphate pathway and tricarboxylic acid cycle by liquid chromatography-mass spectrometry. J Chromatogr A. 2007;1147(2):153–64. 17376459 10.1016/j.chroma.2007.02.034

[26] Li P, Su M, Lämmerhofer M, Chatterjee M. Targeted analysis of sugar phosphates from glycolysis pathway by phosphate methylation with liquid chromatography coupled to tandem mass spectrometry. Analytica Chimica Acta. 2022;1221:340099. 10.1016/j.aca.2022.340099 35934345

